# Global reproductive health – Why do we persist in neglecting the undeniable problem of childlessness in resource-poor countries?

**Published:** 2017-03

**Authors:** W Ombelet, J Goossens

**Affiliations:** Editor-in-Chief; Genk Institute for Fertility Technology, ZOL Hospitals, Schiepse Bos 6, 3600 Genk, Belgium.; The Walking Egg non-profit Organization; Hasselt University, Department of Physiology, Martelarenlaan 42, 3500 Hasselt, Belgium

**Keywords:** Assisted reproduction, childlessness, developing countries, infertility care, public health, reproductive health, reproductive rights, simplified IVF

## Abstract

The consequences of involuntary childlessness in developing countries create more wide-ranging societal problems compared to Western societies, particularly for women. Negative psychosocial and economical consequences for childless couples are often severe and underestimated by the local and international society. Infertility treatment is often limited to certain procedures and certain costumers.

Most common arguments against supporting infertility care in resource-poor countries are the “overpopulation” and the “limited resources” argument, but they totally neglect the reproductive rights and systematic exclusion of millions of women from the right to reproduce.

Because ART procedures are very expensive, governments and international aid-organisations are currently not investing in this technique. But the scene has changed recently: inexpensive ovarian stimulation protocols for IVF have proven their value and simplified but high quality low cost IVF techniques are available nowadays. From an ethical point of view it is our belief that the community can no longer justify the systematic exclusion of one tenth of couples from the right to reproduce in resource-poor countries.

Infertility is a central issue in the lives of many couples who suffer from it. Boivin et al. (2007) described a remarkable similarity in infertility prevalence between 5 and 15 % in all parts of the world, although the reasons for infertility differ substantially. The WHO performed a systematic analysis of 277 health surveys and estimated that worldwide 48.5 million couples are suffering from infertility; half of these couples are living in SubSaharan Africa (SSA) and South Asia (Mascarenhas et al., 2012).

Childlessness and infertility remain an ongoing global challenge, especially for women living in resource-poor settings. The negative consequences of childlessness are more pronounced in developing countries because of different sociocultural circumstances. Childlessness often leads to social isolation, stigmatization, economical deprivation, domestic violence and even suicide (Ombelet, 2008; 2013; Inhorn and Patrizio, 2015).

Infertility is a global health issue and health has been described before as a state of complete physical, mental and social well being and not just the absence of disease. Reproductive health implies an individual’s right to reproduce and this right has been enshrined in the United Nations Declaration of Human Rights, Article 16:1 which states that “Men and women of full age, without any limitation due to race, nationality or religion have the right to marry and found a family” (WHO, 2004).

In this issue of Facts, Views & Vision in Obgyn, Gerrits et al. describe a unique collaboration between different stakeholders to increase knowledge about infertility and childlessness in Ghana and Kenya aiming at generating insight into possible interventions in this field. During their first meeting in Nairobi the audience agreed that policy makers, NGOs and international donor agencies are obliged to recognize the importance and impact of infertility in resource-poor countries (Gerrits et al., 2017).

When looking at the achievements and budgets of most Non-Governmental Organizations (NGOs), foundations and international societies involved in reproductive health, it is clear that the issue of infertility in developing countries is underestimated and neglected not only by the local governments but also by the international non-profit organizations. 

Although it is well known that infertility causes impoverishing health costs as well as economic instability or deprivation secondary to social consequences in resource-poor countries (Dyer and Patel, 2012), the core business of reproductive health care in developing countries is HIV/AIDS, family planning and maternal care and not one single reproductive health care program is dealing with couples unable to reproduce (Dhont, 2012).

There is an urgent need for comprehensive reproductive care initiatives involving maternal and child health, safe abortion, family planning and infertility prevention and management. Why do all these organizations and politicians refuse to tackle the immense medical, economical and sociocultural problems caused by childlessness?

The most cited arguments against supporting infertility care in resource-poor countries are obviously the “overpopulation” and the “limited resources” argument.

The argument of overpopulation suggests that in countries where overpopulation poses a demographic problem, infertility management should not be supported by the government. Therefore, national and international health strategies have always focussed on reducing total fertility rates while infertility care has received little or no attention. Unfortunately high rates of fertility coexist with high rates of infertility in Africa and South Asia, the so-called demographic paradox known as ‘barrenness amid plenty’ (Inhorn and Patrizio, 2015).

On the other hand, many developing countries already succeeded to drop their global fertility rate. United Nations data show that in the majority of developing countries the mean fertility rate has already dropped from more than 5 to 2.6 and is expected to decline to 1.92 by mid-century. Even if infertility treatment including IVF would become more accessible in developing countries it would probably account for less than 2 % of all deliveries. Increasing efforts on family planning and health education should readily overcome this small contribution to the fertility rate especially because family planning project will probably convince more local people if attention is also paid to those who are childless. On the other hand, UN data also show that the expected population growth in developing countries can not solely be attributed to high fertility rates but is mainly due to an improved life expectancy. Life expectancy at birth has increased significantly in the least developed countries in recent years. The six-year average gain in life expectancy among the poorest countries, from 56 years in 2000-2005 to 62 years in 2010-2015, is roughly double the increase recorded for the rest of the world (United Nations, 2015, https://esa.un.org/unpd/wpp/ )

The ‘limited resources’ argument can easily be explained by the scarcity of health resources against a backdrop of limited funds and competing health needs (Okonofua, 1996). In Western circles it is hard to justify expensive fertility treatment in settings with few resources and more important challenges to deal with. We don’t argue that prevention of infertility remains the most cost-effective treatment strategy particularly in countries with a high prevalence of pregnancy-related infections and sexually transmitted diseases (STDs). There is little doubt that prevention is always better than cure. Better education programmes also have proven to be an excellent preventive tool against overpopulation, STDs and pregnancy-related infections. But even with better reproductive health education and preventative care programmes, involuntary childlessness will remain an important problem for millions of couples, as it is the case in Western countries.

But it’s not all about limited resources and overpopulation, other factors also play an important role why funding is lacking. In most developing countries infertility remains a woman’s social burden subsequently leading to less willingness of local authorities to fund infertility care initiatives, women are frequently abandoned to their childless destinies. Moreover, lack of infertility prevention and treatment services is often justified as a strategy for population control especially in high-fertility areas (Inhorn and Patrizio, 2015).

Most cases of infertility in developing countries can only be treated with assisted reproductive technologies due to the high rate of tubal block and declined sperm quality as a result of infections. Consequently, treatment is restricted to the happy few who can afford it unless we succeed in making these ART techniques accessible by simplifying them substantially. Mild low cost ovarian stimulation protocols have been studied recently, with very promising results (Ferraretti et al., 2015). Moreover, simplified low cost IVF techniques have been developed with very good results as well (Ombelet, 2014; Van Blerkom et al., 2014). With the latter becoming available, there should be no impediment for infertility care to become integrated into mainstream reproductive health care in developing nations anymore (Dhont, 2012). 

It is our experience that most infertility experts from resource-poor countries are not really supporting the idea of “accessible infertility care” including simplified IVF. Due to the lack of experienced medical doctors it remains very difficult to implement this idea of affordable IVF in sub-Saharan Africa. Local politicians and health care providers, influenced by the local IVF lobbies, hesitate to financially support these projects despite the high demand of patient organizations.

During the past 10 years we experienced that almost all international organizations involved in reproductive health care acknowledge the importance and consequences of infertility and childlessness in resource-poor countries, but they still exclude infertility care as a possible goal for future projects. According to Dhont (2012) the neglect of infertility in the public health debate is caused by a mixture of ignorance (mainly by the international aid community) and tunnel vision, opportunism and a non-enlightened attitude of contempt for individual human rights.

But do we have the will to act, financially and politically? We actually have the means to provide accessible infertility care to a large part of the world population. Action is urgently needed and is the responsibility of all actors involved: governments in developing countries, NGOs, the women’s health movement, philanthropic foundations, the health profession and the research community.

We sincerely hope and strongly believe that if affordable solutions become operational, infertility care will be integrated into mainstream reproductive health care in resource-poor countries. This achievement has the potential to give dignity not only to more than 20 million “neglected” couples but also to give dignity to “distinguished” reproductive health care programs and organisations (Dhont, 2012).
